# Intestinal cryptosporidiosis in HIV-infected patients in the department of infectious diseases

**DOI:** 10.1186/1471-2334-14-S2-P52

**Published:** 2014-05-23

**Authors:** L Badoui, G Dabo, H Lamdini, A Oulad Lahcen, M Sodqi, L Marih, A Chakib, M Soussi, K Marhoum El Filali

**Affiliations:** 1 Ibn Rochd University Hospital, Casablanca, Morocco

## Introduction

The Cryptosporidiosis is a gastrointestinal opportunistic parasitic disease is a major cause of diarrhea and malnutrition in patients infected with HIV. Its prevalence is estimated at 2.76%. The aim was to determine the prevalence of intestinal Cryptosporidium in patients infected with HIV and to identify Cryptosporidium species in question.

## Materials and methods

This is a retrospective study conducted 20 months in the department of Infectious Diseases. Cryptosporidium research was conducted by the Ziehl-Neelsen and modified by PCR. Data collection was made from computerized records.

## Results

We collected 75 patients. The mean age of patients was 37 years old. The median CD4 was 62cel/mm ³. Intestinal cryptosporidiosis was indicative of HIV infection in 54 cases (77%) and in 16 cases (23%) they occurred at the waning of treatment failure. Cryptosporidia were isolated from 52 patients (69.3%). Eight of them were gay. Consumption of raw milk and the presence of animals in the environment have been reported by more than two thirds of patients. Thirty five infected individuals were diarrhea, 12 had febrile abdominal pain. Cryptosporidium parvum was the species most frequently isolated. 80% of patients had progressed well (n = 60), and we recorded 20% of deaths (n = 15).

## Conclusion

The prevalence of cryptosporidiosis is higher in patients infected with HIV. The strengthening of preventive measures is necessary especially as some of the identified species at risk of human transmission in hospitals. The prognosis of the disease remains in the absence of effective therapy, dark in refractory cases symptomatic treatment and HAART.

